# Effects of Diets Enriched in Omega-9 or Omega-6 Fatty Acids on Reproductive Process 

**Published:** 2016-06

**Authors:** Seyedeh Neda Mousavi, Fariba Koohdani, Farzad Shidfar, Leila Shafiei-Neek

**Affiliations:** 1 Department of Biochemistry and Nutrition, School of Medicine, Zanjan University of Medical Sciences, Zanjan, Iran; 2 Department of Cellular and Molecular Nutrition, School of Nutritional Sciences and Dietetics, Tehran University of Medical Sciences, Tehran, Iran; 3 Department of Nutrition, School of Health, Iran University of Medical Sciences, Tehran, Iran; 4 Department of Sport Physiology, School of Physical Education and Sport Sciences, University of Tehran, Tehran, Iran

**Keywords:** Gestation, Sex Ratio, Offspring, Dietary Oil

## Abstract

**Objective:** Maternal type and amounts of dietary fatty acids affect on reproductive process in the mice. The present study investigated the effects of maternal supplementation with different amounts of omega-6 or omega-9 during pregnancy on the number of offspring, sex-ratio and duration of gestation.

**Materials and methods:** Eight-week-old female C57BL/6 mice were randomly assigned into four dietary groups including low omega-6 (16%; LO6), low omega-9 (16%; LO9), high omega-6 (45%; HO6) and high omega-9 (45%; HO9) during gestation. Number of offspring, sex-ratio and duration of pregnancy were compared among four dietary groups.

**Results:** There was significant difference between LO6 and HO6 (p < 0.0001), LO9 and HO9 (p < 0.0001) groups in total number of pups. The number of female and male offspring were significantly different between LO6 and LO9 (p = 0.009 and p = 0.001, respectively), LO9 and HO9 (p = 0.01 and p = 0.025) groups. Duration of pregnancy was significantly higher in low fat diet than high fat diet groups (< 0.001).

**Conclusion:** High fat diet reduced number of pups, gestation duration and lead to early labor. Omega-9 fatty acids shifted sex of offspring to females.

## Introduction

Maternal nutrient supply (quantity and/or quality) can influence on fertility, duration of pregnancy and birth of a healthy offspring (1). Fats as an important component of energy in diet can lead to adverse outcomes for mothers and infants by affecting on reproductive process (2). Fatty acids (FAs), mainly essential FAs and long-chain polyunsaturated fatty acids (LC-PUFAs) have two or more double bonds per FA (PUFAs), which are the main characteristic of the Western Style Diet (WSD) (3, 4). Vegetable oils are the main sources of omega-6. They are including soy, sunflower and corn oils. Omega-9 fatty acids (MUFAs) have one double bond per FA. Oleic acid (18:1 n-9), as the major MUFAs, is the main characteristic of the Mediterranean Style Diet (MSD) which is high in olive oil (5). Many studies have reported that diet enriched with saturated or unsaturated fatty acids can alter serum steroid concentrations in some species including rodents, food animals and humans (6). PUFA supplementation increased duration of pregnancy, lead to difference in onset of labor (7), altered fatty acid composition of ovarian follicle and increased follicle number and size in cattle (8), also ovulation rate increased in mice (9). *In vitro* culture of linoleic acid resulted in impairment of embryo development (10). PUFAs are essential for the energy source and cellular functions of the oocyte.Changes in the fatty acid composition or its level in the diet alters the fatty acid content of oocyte and its surrounding environment, having an impact on oocyte maturation and developmental competence at the term of fertilization and embryo viability.PUFAs stimulate the generation of reactive oxygen species (ROS) in vitro and have a physiologic effect on oocyte and embryo development (11). Studies have shown that omega-3 fatty acids increase apoptosis in colonocytes due to oxidative stress (12). Numerous studies have shown that PUFAs lead to increase in mitochondrial synthesis and oxidation which is correlated with organization (13, 14). Another suggested mechanism is that free fatty acids and lipid accumulation lead to structural alterations which impair endoplasmic reticulum (ER) functions (15). Mitochondrial damage may cause more lipid accumulation byimpairing in lipid catabolize and ultimately apoptosis (16), then excess lipid in diet lead to lipid-induced programmed cell-death (17). In another hand, previous studies in opossums and humans suggest that females on a diet supplemented with PUFA give birth to more boys than girls (18). To our knowledge, no animal studies have been performed to study the concurrent effects of type and amount of maternal dietary oils in an isocaloric diet after conception on the number of offspring, sex-ratioand duration of pregnancy in mouse. So, the present study was designed to determine whether changes in type and/or amount of maternal fatty acid status during pregnancy with similar energetic values had consequences on the number of litters, sex-ratio in the offspring and duration of pregnancy in mothers.

## Materials and methods

***Animals***

Animal experiment was approved by the Animal Research Committee of Tehran University of Medical Sciences. This research conforms to the Institutional and National Guide for the Care and Use of Laboratory Animals. Forty, Eight-week-old female C57BL/6 mice (21 ± 1.5 g) were obtained from Razi Vaccine and Serum Research Institute, Tehran, Iran. Each mouse was individually housed at 21–23 °C and controlled humidity (50 ± 5%) under a 12 h artificial light cycle (7 am to 7 pm). All animals received AIN93M diet two weeks before beginning of the study for adaptation. 

***Diets***

Diets containing specific fatty acids (omega-9 and omega-6 diets) with different amounts were prepared as described in the table 1. Low Omega-6 diet (LO6) containing 20% of energy as protein, 64% of energy as total carbohydrates (10% sugar, 54% corn starch) and 16% of energy as soy oil. High omega-6 group (HO6) containing 20% of energy content as protein, 35% of energy as total carbohydrates (10% sugar, 25% corn starch) and 45% of energy as soy oil. Low Omega-9 (LO9) and high omega-9 diets (HO9) have the same content except the type of oil (Extra Virgin Olive Oil). All diets were isocaloric and contained 3.97 kcal/gr by dry weight. The standard diet composition (per 1 kg of diet) was 200 g of protein as Casein lactate (Iranian Caseinate Industry) and 3 gr as L-cystein (W326305, Sigma), 70 gr of lipid as soy oil or EVOO (Kamzit Company, Iran), 530 gr of carbohydrate as corn starch (corn dextrin from corn refinery) and 100 gr as sucrose, 35 g of AIN93G mineral mix (296040002, MP biomedicals), 10 g of AIN93 vitamin mix (296040201, MP biomedicals), 2.5 gr of choline bitartrate (C1629, Sigma), 0.008 gr as tert-butyl hydroquinone (112941, Sigma) and up to 100 g of fiber (wheatbran) (Table 1).

***Breeding and measurements***

Each female housed individually and were mated with one male per cage overnight. After vaginal plaque confirmation, mothers were randomly assigned to four different dietary groups. Animals were fed with one of the four isocaloric balanced diets (paire-fed model) (19) during pregnancy.

Animals weighed on a calibrated balance scale to the nearest 0.1 gr weekly for the determination of weight changes. 

***Statistical Analysis***

To adjust for potentially confounding variables and assess the interactions between different variables two-way ANOVA were used.

**Table 1:** Composition of the experimental diets per 1 kg during the study (AIN93G diet)

**Diets**	**LO6**	**LO9**	**HO6**	**HO9**
**Nutrients (g/kg)**				
Casein (gr)	200	200	200	200
Cornstarch (gr)	530	530	247	247
Sucrose (gr)	100	100	100	100
Soy oil (gr)	70	-	198	-
EVOO (gr)	-	70	-	198
Fiber (gr)	50	50	204.5	204.5
Mineral mix (gr)	35	35	35	35
Vitamin mix (gr)	10	10	10	10
L-cys (gr)	3	3	3	3
Choline bitartrate (gr)	2.5	2.5	2.5	2.5
tert-butyl hydroquinone (gr)	0.008	0.008	0.008	0.008
Energy (Kcal/gr)	3.97	3.97	3.97	3.97
As carbohydrate	64%	64%	35%	35%
As fat	16%	16%	45%	45%
As protein	20%	20%	20%	20%

LO6: Low Omega-6 diet, LO9: Low Omega-9 diet, HO6: High Omega-6 diet, HO9: High Omega-9 diet; EVOO: Extra Virgin Olive Oil, L-cys: L-cystein

This test was performed to assess the effect of type and amount of oils on maternal weight gain, birth weight of offspring, as well as number of pups, sex of offspring and duration of pregnancy. Whenever interaction was significant, the main effect of factors was evaluated in subgroups. For comparison of continuous variables in two groups we used independent samples t-test and to compare more than two groups one way ANOVA was performed. All data were expressed by means ± SD. The level of significance was considered at P < 0.05. Statistical analyses were performed with IBM SPSS Statistics software (version 20; IBMCorp).

## Results

***Maternal and offspring weight***

Maternal dietary intake (energetic value) was not different among four dietary groups (p > 0.05). Results showed that the interaction between type and amount of dietary fatty acids on maternal weight gain was not significant during three weeks of gestation. As shown in table 2, the main effect of omega-6 on maternal weight gain, adjusted for the amount of dietary fatty acids, was significantly higher than omega-9 in all three weeks of gestation (p < 0.05). Except the 1^st^ week of gestation, the main effect of low fat diet group on maternal weight gain was significantly higher than high fat diet group (p < 0.05). Obtained results showed that the interaction between type and amount of dietary fatty acids on birth weight of female offspring were not significant. The main effect of omega-6 on birth weight, adjusted for the amount of dietary fatty acids, was significantly higher than omega-9 diet group (p < 0.001). The interaction between type and amount of dietary fatty acids on weight was not significant at adolescence. The main effect of omega-6 on weight was significantly higher than omega-9 at adolescence, adjusted for the amount of dietary fatty acids (p < 0.001). Adjusting for type of dietary fatty acids, the main effect of amount of dietary fatty acids on weight at adolescence was significantly higher in low fat than high fat diet group (p = 0.001). In male offspring, the interaction between type and amount of dietary fatty acids on birth weight were not significant. The main effect of omega-6 on birth weight, adjusted for the amount of dietary fatty acids, was significantly higher than omega-9 (p < 0.001). The interaction between type and amount of dietary fatty acids on weight was not significant at adolescence. The main effect of omega-6 on weight was significantly higher than omega-9 at adolescence, adjusted for the amount of dietary fatty acids (p < 0.001). Adolescence weight was significantly higher in high fat than low fat diet groups, Adjusted for type of dietary fatty acids (p = 0.02).

**Table 2:** Effects of type and amounts of maternal dietary fatty acid on mother' weight gain during pregnancy, weight of offspring at birth and pregnancy duration

Variables	Omega-6	Omega-9	Low fat (16%)	High fat (45%)
**Maternal (n = 10)**				
**Weight gain at week 1 (gr)**	**22.1 ± 1.02**	**21.3 ± 0.9**	**21.1 ± 0.7**	**22.3 ± 0.9**
**P value** †	**0.002** *		**< 0.001** *	
**Weight gain at week 2 (gr)**	**35.6 ± 2.6**	**30.8 ± 2**	**34.9 ± 3.2**	**31.4 ± 2.4**
**P value** †	**< 0.001** *		**< 0.001** *	
**Weight gain at week 3 (gr)**	**40.7 ± 2.4**	**37.9 ± 1.8**	**40.7 ± 1.7**	**38 ± 2.5**
**P value** †	**< 0.001** *		**< 0.001** *	
**Female offspring (n = 10)**				
**Birth weight (gr)**	**1.29 ± 0.2**	**1 ± 0.09**	**1.13 ± 0.3**	**1.16 ± 0.2**
**P value** †	**< 0.001** *		**0.63**	
**Male offspring (n = 10)**				
**Birth weight (gr)**	**1.5 ± 0.28**	**1.07 ± 0.13**	**1.4 ± 0.4**	**1.2 ± 0.15**
**P value** †	**< 0.001** *		**0.01** *	
**Duration of pregnancy (day)**	**20.8 ± 1.4**	**20.6 ± 1.1**	**21.8 ± 0.9**	**19.7 ± 0.54**
**P value** †	**0.35**		**< 0.001** *	

Values are reported as mean ± SD

† Two way ANOVA was used,

* statistically significant

***Number of pups, sex ratio and duration of pregnancy***

The interaction between type and amount of dietary fatty acids on duration of pregnancy was not significant. The main effect of omega-6 and omga-9 fatty acids on duration of pregnancy was not significantly different, adjusted for amount of dietary fatty acids. Adjusting for type of dietary fatty acids, duration of pregnancy was significantly higher in low fat diet than high fat diet group (p < 0.001) (Table 2).

The interaction of type and amount of dietary fatty acids on sex ratio and number of pups was significant (p < 0.001 and p < 0.001, respectively). The subgroup analysis of these variables is showed in table 3. There was a significant difference among four dietary groups (p < 0.0001) and also, between LO6 and HO6 (p < 0.0001), LO9 and HO9 (p < 0.0001) groups in total number of pups. The number of male offspring were significantly different between LO6 and LO9 (p = 0.001), LO6 and HO6 (p < 0.0001), LO9 and HO9 (p = 0.025). The number of female offspring were significantly different between LO6 and LO9 (p = 0.009), LO9 and HO9 (p = 0.01) groups, but not significant between LO6 and HO6 groups (p = 0.054). No significant difference was shown between HO6 and HO9 groups in terms of the number of pups and also sex ratio in offspring. Duration of pregnancy was significantly different between LO6 and HO6 (p < 0.0001) and also, between LO9 and HO9 groups (p < 0.0001). There was no significant difference between HO6 and HO9 groups.

**Table 3:** Effects of type and amounts of maternal dietary fatty acid on total number and sex ratio of off spring

** Diets**	**LO6**	**LO9**	**HO6**	**HO9**	**P value** †
**Variables**					
Total pups	76^a^	74^b^	39^a^	37^b^	< 0.001*
N. of male	49^a^, ^c^	29^b^, ^c^	26^a^	15^b^	< 0.001*
N. of female	27^c^	45^c^, ^d^	13	22^d^	< 0.001*
(%) Male	64.4	39.2	66.6	40.5	
(%) Female	35.6	60.8	33.4	59.5	

LO6: Low Omega-6 diet, LO9: Low Omega-9 diet, HO6: High Omega-6 diet, HO9: High Omega-9 diet

† One- way ANOVA was used,

* Statistically significant

a : Comparison of LO6 and HO6 groups valued by post-hoc test (p < 0.0001 in male and females);

b : Comparison of LO9 and HO9 groups valued by post-hoc test (p < 0.0001 in females and p = 0.025 in males);

c : Comparison of LO6 and LO9 groups valued by post-hoc test (p = 0.009 in females and p = 0.001 in males);

d : Comparison of LO9 and HO9 groups valued by post-hoc test (p = 0.01)

## Discussion

In the present study, type and amount of dietary fatty acids had significant effect on maternal weight gain and weight of offspring at birth, both in males and females. The number of pups was significantly lower in high oil than low oil groups, both in omega-6 and omega-9 groups. The number of female offspring was higher in omega-9 than omega-6 group. The number and sex-ratio were not significantly different between high omega-6 and high omega-9 diets. Amount of dietary oils, but not type of fatty acids, had significant effect on duration of pregnancy. High dietary oils lead to decrease in gestation period and early delivery. To our knowledge, this is the first animal study to compare the effects of maternal diet enriched with different amounts of omega-6 and omega-9fatty acids,in an isocaloric diet, on number of pups, sex ratio and gestation duration. We speculated that type and amount of fatty acids after conception, may alter cervical mucus, vaginal PH and change the sex-ratio and number of pups.Analysis of preterm mortality records from the Medical Registry in Norway revealed that human male embryos appear to be more sensitive to uterine stress and likely aborted than females (20).Pups of the omega-9 fed mice had slow weight gain after birth. One possibility is the quality and/or quantity of milk that produced by mothers, mammary duct development or suckling response of pups. Low amounts or quality of milk lead to failure to thrive (21). High fat diet is a nutritional stress for mothers. Then, reduction in the number of pups and male offspring is expected (22). Alteration in number of offspring may be due to change in fatty acid composition of the oocyte and its surrounding environment which effect on oocyte maturation, developmental process, fertilization ability and embryo viability. Several reports have indicated that in utero or perinatal omega-supplemented diets alter behavioral responses, growth and metabolic hormones in offspring (23, 24). Some studies have shown that fat composition of diet, especially the fatty acids, can influence numerous aspects in the reproductive process, including oocyte maturation and timing of ovulation (25), the production of chemo-attractants by oocytes (26), prostaglandin synthesis and properties of the reproductive tract (27), which effect on the fertilization abilities and sex-ratio (28). In one study, effects of diets containing omega-3 and omega-6 PUFAs were assessed on offspring sex-ratio and maternal behavior in mice (22). In comparison to omega-3 group, mothers fed omega-6 containing diet birthed more daughters than sons and had more anxiety. In this study, corn oil-enriched N-6 diet increased female ratio to 60%. Also, fish oil-enriched N-3 diet had no effect on sex-ratio, this group had equal number of male and female offspring born and weight gain of dams were not significantly different. This study was not a pair-fed model and they couldn't measure food consumption in dietary groups. These results are inconsistent with our pair-fed study, which N-9 diet increased female ratio to 60% and also, maternal weight gain was significantly different among four dietary groups and also, between low omega-6 and low omega-9 groups.Our study compared soy oil-enriched N-6 diet and EVOO-enriched N-9 diet. Type and amounts of dietary fatty acids are different from mentioned study. Rivers and Coward (29) fed mice either a low fat or control diet. Females on the low fat diet had litters with a significant sex-ratio distortion (1:3, male: female). This means that there had been selective loss of male embryos due to a decrease in uterine glyceryl-phosphoryl choline diesterase activity (30). Another speculated mechanism is that PUFA exposure may implicate the process of fertilization by Reactive Oxygen Species (ROS) production. ROS are important cytotoxic signaling molecules in the cell which are generated by lipid peroxidation and are considered important in many cell pathways. Fatty acids are the main source of energy for oocytes during maturation (31) and a change in the fatty acid profile of the ovary potentially alters the energy source of the developing oocyte. 

In the previous studies, the effects of maternal dietary saturated fatty acids, omega-6 and omega-3 were assessed in peri-conceptional period (32). This was the first study to assess concurrent effect of amount and type of maternal dietary fatty acids after conception on sex-ratio, number of offspring and duration of pregnancy with focus on omega-9.More studies are needed to understand the underlying mechanisms for these effects. This study conclude that fatty acid composition of maternal diet after conception influence on the reproductive process, oocyte maturation, sex ratio, number of pups and timing of pregnancy. High fat diet reduced the number of pups and early delivery. Also, type of fatty acids affect on the sex ratio. Omega-9 diet shifted the offspring to more female than male. In addition to calorie content, type and amount of dietary fatty acids can play a directive role in this process.
